# Melatonin enhanced low-temperature combined with low-light tolerance of pepper (*Capsicum annuum* L.) seedlings by regulating root growth, antioxidant defense system, and osmotic adjustment

**DOI:** 10.3389/fpls.2022.998293

**Published:** 2022-09-28

**Authors:** Jing Li, Jianming Xie, Jihua Yu, Jian Lyv, Junfeng Zhang, Dongxia Ding, Nenghui Li, Jing Zhang, Emily Patience Bakpa, Yan Yang, Tianhang Niu, Feng Gao

**Affiliations:** ^1^ College of Horticulture, Gansu Agricultural University, Lanzhou, China; ^2^ Institution of Vegetable, Gansu Academy of Agricultural Science, Lanzhou, China

**Keywords:** pepper, melatonin, root morphology, reactive oxygen species, antioxidant defense system, ascorbate-glutathione cycle, osmotic adjustment

## Abstract

Melatonin (MT) is an important biologically active hormone that plays a vital role in plant growth and development. In particular, it has been investigated for its roles in abiotic stress management. In this study, pepper seedlings were subjected to low-temperature combined with low-light stress (LL) (15/5°C, 100 μmol m^-2^s^-1^) prior to a foliar spray of 200mM MT for 168h to investigate the protective role of MT in pepper seedlings. Our results demonstrated that LL stress negatively affected root growth, and accelerated the accumulation of reactive oxygen species (ROS), including H_2_O_2_ and ${\text{O}}_{2}^{-}$ , changed the osmolytes contents, and antioxidative system. However, these were reversed by exogenous MT application. MT effectively promoted the root growth as indicated by significant increase in root length, surface area, root volume, tips, forks, and crossings. In addition, MT reduced the burst of ROS and MDA contents induced by LL, enhanced the activities of SOD, CAT, POD, APX, DHAR, and MDHAR resulted by upregulated expressions of *CaSOD*, *CaPOD*, *CaCAT*, *CaAPX*, *CaDHAR*, and *CaMDHAR*, and elevated the contents of AsA and GSH, declined DHA and GSSH contents, which prevented membrane lipid peroxidation and protected plants from oxidative damages under LL stress. Furthermore, seedlings treated with MT released high contents of soluble sugar and soluble protein in leave, which might enhance LL tolerance by maintaining substance biosynthesis and maintaining cellular homeostasis resulted by high levels of osmolytes and carbohydrate in the cytosol. Our current findings confirmed the mitigating potential of MT application for LL stress by promoting pepper root growth, improving antioxidative defense system, ascorbate-glutathione cycle, and osmotic adjustment.

## 1 Introduction

The growth of pepper (*Capsicum annuum* L.) is closely related to suitable external conditions including light, temperature, water, gas, fertilizer and other factors (Eom et al., 2010). Pepper is a typically thermophilous and heliophilic vegetable that originated from South Africa. It is mainly cultivated in protected facilities out of season during the winter-spring season, especially in northwest China. Due to the limited heat preservation performance and poor controllability of solar greenhouses, temperature and light are often difficult to reach the level needed for growth and development of pepper (Ou et al., 2015; Li et al., 2021; Tang et al., 2021). Low-temperature combined with low-light stress (LL) not only seriously affects the yield and quality of pepper, but also decreases the production and management costs.

Chilling stress decrease the plant root growth by decreasing the biomass of the roots, and this root growth inhibition is contributed to the constrain of cell elongation and differentiation shape of root cells (Nagasuga et al., 2011; Zhu et al., 2015; Yamori et al., 2022). In contrast, light regime affects the root distribution, and low light leads to longer but less branched roots in plants (Page et al., 2012). Besides, the cell membrane is the first place where plants are damaged by low-temperature stress. Low-temperature stress leads to changes of the membrane phase, decreased fluidity, and increased cell membrane permeability, eventually causing ion metabolism disorders as a result of cytoplasmic exudation (Barrero-Sicilia et al., 2017). In addition, under low-temperature stress, reactive oxygen species (ROS) react with unsaturated fatty acids in the cell membrane to produce malondialdehyde (MDA) (Dey et al., 2021). The decreased of ROS scavenging ability of plants under low-temperature stress leads to the production of a large number of MDA, and finally changes the structure and function of membrane proteins (Lado et al., 2016; Chen et al., 2018). Under stress, plants will activate the enzymatic defense system, which can significantly increase the activities of defense enzymes such as superoxide dismutase (SOD), peroxidase (POD), catalase (CAT), ascorbate peroxidase (APX), glutathione reductase (GR), dehydroascorbate reductase (DHAR), monodehydroascorbate reductase (MDHAR), and ascorbate-glutathione (AsA-GSH) cycle and key genes expressions, and eliminate excess free radicals to protect from the injury caused by stress (Zhao et al., 2016). In addition, light intensity directly affects plant growth characteristics and plant metabolism. Weak light causes a significant increase in ROS, which in turn leads to an increase in MDA, resulting in changes in the antioxidant activity of a series of enzymes, and osmotic regulation (Meng et al., 2017; Mclay et al., 2018). However, studies on the effects of low-temperature combined with low-light stress on the root and antioxidant defense system of pepper plants are not available.

Melatonin (N-acetyl-5**-**methoxytryptamine, MT) is an indole hormone with multiple functions and is ubiquitously present in animals, plants, and algae (Hattori et al., 1995). Endogenous MT plays a vital role in plants, such as regulating seed germination, plant growth, root system architecture (root elongation, adventitious root formation, and lateral development), and circadian rhythm (Park and Back, 2012; Zhang et al., 2013; Balabusta et al., 2016). Additionally, MT acts as an endogenous oxygen free radical scavenger that can eliminate excessive ROS caused by stress, improve the activity of antioxidant enzyme and the regulation ability of the ASA-GSH system, and inhibit the content of H_2_O_2_ and ${\text{O}}_{2}^{-}$ in plants protecting plants from oxidative damage (Zhang N. et al., 2015; Xu et al., 2016; Chen et al., 2017; Li et al., 2019a; Moustafa-Farag et al., 2020; Zeng et al., 2022). Numerous studies have proved the antioxidative effect of MT on rice, cucumber, tomato, maize, *Coffea arabica* L., and *Camellia sinensis* L. (Debnath et al., 2018; Li et al., 2018; Wei et al., 2018; Ahammed et al., 2019; Ahmad et al., 2019; Campos et al., 2019; Ahammed et al., 2020; Wang et al., 2020; Zhang T. et al., 2020; Yan et al., 2021). Therefore, MT regulates the metabolic process of plants to improve plant resistance, and effectively alleviate many stresses such as low temperature, high temperature, drought, and salinity (Li et al., 2017; Zhang et al., 2017a; Sharma and Zheng, 2019; Ren et al., 2020; Wang et al., 2020; Imran et al., 2021).

Although much distinct progress has been made in exploring the role of MT in response to abiotic stress, little is known about the mechanism of MT on LL tolerance in pepper. In this study, the impacts of exogenous MT pre-application on the root structure, H_2_O_2_, ${\text{O}}_{2}^{-}$ , and MDA contents; soluble sugar and soluble protein contents; as well as activities of SOD, POD, CAT, APX, DHAR, MDHAR, and contents of AsA, DHA, GSH, GSSH of pepper seedlings under LL stress were investigated to elucidate the possible mechanism of MT involved in response to LL.

## 2 Materials and methods

### 2.1 Plant materials, and growth conditions

Two geminated pepper seeds (*Capsicum annuum* L.) cv. ‘Hangjiao 4’ (purchased from Tianshui Academy of Agricultural Sciences) were sown in substrate (vermiculite: grass carbon: cow dung = 3:1:1). The growth conditions were maintained at 28°C/18°C (day/night) temperature with relative humidity of 70%-80% under 12h photoperiod (300μmol m^–2^ s^–1^) in the greenhouse of Gansu Agricultural University (N36°05’39.86’’, E103°42’31.09’’). The plants were watered every day, and fertilized with 1/2 Hoagland nutrient solution once a week.

### 2.2 Treatments

Pepper seedlings at the five-leaf stage were selected for stress treatments. Four treatments were conducted in this study including (1) CK, 28 °C/18 °C, 300 μmol m^-2^ s^-1^, distilled water; (2) CK+MT 28 °C/18 °C, 300 μmol m^-2^ s^-1^, 200 μM MT; (3) LL, 15°C/5°C, 100 μmol m^-2^ s^-1^, distilled water; and (4) LL+MT, 15 °C/5 °C, 100 μmol m^-2^ s^-1^, 200 μM MT. The melatonin (Sigma-Aldrich, USA) was dissolved in little ethanol followed by dilution with distilled water [ethanol/water (v/v) = 1/10,000]. All solutions contained 1% Tween 80 and each spray was carried out at 8:00 pm for four continuous days, while control seedlings were instead of equal amounts of distilled water. After that, the seedlings were transferred to an artificial climate incubator (RDN-400E-4, Zhejiang, China) for different temperature and light stresses. The third expanded pepper leaves were collected randomly, then frozen in liquid nitrogen, and kept at -80 °C for physiological and biochemical analysis after 0, 6, 12, 24, 48, and 168h of the treatment. Each treatment was repeated three times with 30 plants per replicate.

### 2.3 Root structure

The root structure was scanned by using a root scanner (Epson). The root length, root surface area, root volume, tips, forks, and crossing of pepper seedlings treated for 168h were determined using WinRHIZO software (LC4800-II LA2400, Sainte Foy, Canada) (Zhang et al., 2013).

### 2.4 Histochemical staining and analyses of ROS and MDA content

Superoxide anion ( ${\text{O}}_{2}^{-}$ ) was detected visually by treating leaves with nitro blue tetrazolium (NBT) after 168h as described by Tang et al. (2021). Pepper leaves during five-leaf stage of 0.1g grounded into powders with liquid nitrogen was used to measure ${\text{O}}_{2}^{-}$ and H_2_O_2_ content in accordance with the instructions of reagent kits after 0, 6, 12, 24, 48, and 168 h (Suzhou Keming Biotechnology Co., Ltd, China). Finally, the absorbances of samples were measured at 530nm and 415nm, respectively. The MDA content was determined using the thiobarbituric acid method *via* detecting the absorbance at 450, 532, and 600nm according to the method of Kong et al. (2014).

### 2.5 Analyses of osmotic adjustment substances

Soluble sugar was determined using the anthrone method based on a precious study (Shi et al., 2015). In brief, 0.1g of fresh pepper leaves was added to 2mL of 80% ethanol, and boiled for 30 min. Then 100μL of the extracts was added to 2mL anthrone, followed by boiling water bath for 10min. The absorbance of the above mixture was measured at 620 nm wavelength with a spectrophotometer. Total soluble proteins were determined with the Bradford reagent. The absorbance at 595 nm was recorded, and the soluble protein level was calculated according to the standard curve (Bradford, 1976).

### 2.6 Extractions and assays of antioxidant enzymes

#### 2.6.1 Activity of SOD, POD, and CAT

About 0.5g of fresh leaves and 0.1 g of quartz sands were homogenized in 5.0 mL of 50mM phosphate buffer (pH 7.5) containing 0.1mM ethylene diamine tetraacetic acid and 5.0% (w/v) polyvinylpyrrolidone with a cold mortar. Then they were transferred into centrifugal tubes, and centrifuged at 15,000g for 15 min under 4°C. The supernatants were used for measurements of SOD, POD, CAT. All of these antioxidant enzymes were assayed according to the instruction of the reagent kits (Suzhou Keming Biotechnology Co., Ltd, China). The activities of enzymes were determined by observing at 470, 560, and 240 nm, respectively.

#### 2.6.2 Determination of ASA-GSH cycle contents and key enzymes

The contents of antioxidant, including reduced ascorbic acid (AsA), dehydroascorbate (DHA), reduced glutathione (GSH) (Kong et al., 2014), and oxidized glutathione (GSSG), and the activities of key enzymes in AsA-GSH, including APX, MDHAR, DHAR were determined by using reagent kits purchased from Suzhou Keming Biotechnology Company (China).

### 2.7 Data validation and gene expression analysis by quantitative real-time polymerase chain reaction (qRT-PCR)

The transcript levels of genes, including *CaSOD*, *CaPOD*, *CaCAT*, *CaAPX*, *CaDHAR*, and *CaMDHAR*, in pepper leaves were determined by qRT-PCR. Total RNA from fresh pepper leaves was extracted by using an RNAprep Pure Plant kit (Tiangen Biotech Co., Ltd., Shanghai, China). The RNA quality and concentration were detected by Nanodrop 2000 (Thermo, MA, USA). RNA of 0.5 micrograms was reverse-transcribed into cDNA using the cDNA Synthesis Kit (Takara Biotech Co., Ltd., Dalian, China). Oligo7 software was used to design primers, and the primer sequence of target genes is provided in **Table S1**. Finally, qRT-PCR was performed using the TransStart Green qPCR SuperMix reagent Kit (TransGen Biotech, Beijing, China) on a LightCycler 480 II system (Roche, Switzerland). The reaction conditions include 30 s preincubation at 94°C, followed by 40 cycles at 95°C for 5 s, 60°C for 30 s, and 72°C for 15 s. Each sample was measured with three technical replicates. Actin was used as an internal reference gene and the relative expressions of the relative expressions of target genes were analyzed using the 2^-ΔΔCt^ method (Livak and Schmittgen, 2001).

## 3 Statistical analysis

All experiments were conducted using three biological repeats. Duncan’s multiple comparison test at a significance level of *P* < 0.05 was carried out using SPSS19.0 software (IBM SPSS 22.0, IBM Corporation, New York, USA) for statistical analysis. The figures were generated in Origin ver. 8.5 and Adobe Photoshop CS 6.

## 4 Results

### 4.1 Effect of exogenous MT on root morphology and growth in pepper under LL stress

**Figure 1:**
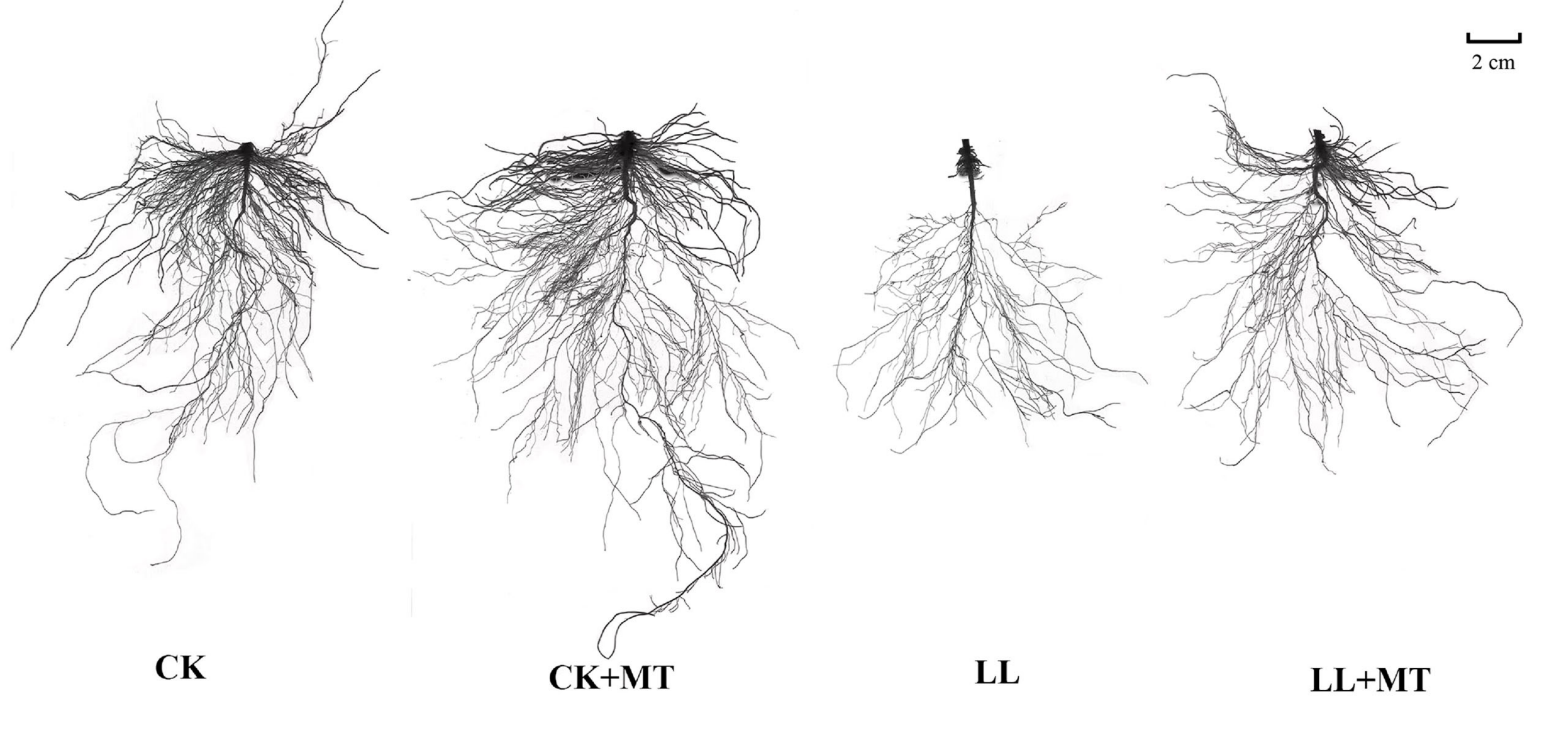
Effect of exogenous MT on the root morphology in pepper under LL stress. CK (normal temperature and light, 28°C/18°C and 300 μmol photons m^-2^ s^-1^), CK+MT (normal temperature and light, 28°C/18°C and 300 μmol photons m^-2^ s^-1^, 200 μmol L^-1^ melatonin), LL (low-temperature combined with low-light, 15°C/5°C and 100 μmol m^-2^ s^-1^), and LL+MT (low–temperature combined with low-light, 15°C/5°C and 100 μmol m^-2^ s^-1^, 200 μmol L^-1^ melatonin). Vertical bars represent the standard errors of the means (three replicates. Different letters denote statistically significant differences among treatments within same time at a level of *P* < 0.05 by Duncan’s multiple comparison tests.

After 168 h, no significant changes in root growth of pepper seedlings were observed under CK and CK+MT; however, LL stress conditions severely hampered the root growth, which was reversed by MT pre-spraying (**Table 1** and **Figure 1**). The root length, surface area, root volume, tips, forks, and crossing of pepper seedlings pre-sprayed with MT showed no significant changes under normal conditions compared with those under CK. However, LL stress remarkably reduced the root length, surface area, root volume, tips, forks, and crossing of pepper seedlings by 58.14, 69.08, 76.67, 42.14, 76.45, and 74.85%, respectively, compared with CK. Compared with LL, the root volume, root length, surface area, tips, forks, and crossing were increased by 69.55, 105.25, 142.86, 67.60, 142.92, and 164.75%, respectively, after pre-application of MT. Except the root volume, all parameters reached significant levels (*P* < 0.05).

**Table 1:** Effect of exogenous MT on the root growth in pepper under LL stress.

Treatment	Length (cm)	Surface Area (cm^2^)	Root Volume (cm^3^)	Tips	Forks	Crossings
**CK**	539.87 ± 42.05a	77.64 ± 11.45a	0.90 ± 0.20a	480 ± 35.51a	2760 ± 476.30a	586.5 ± 83.12a
**CK+MT**	558.57 ± 37.00a	80.48 ± 6.29a	0.93 ± 0.09a	563.25 ± 48.32a	2711 ± 328.59a	568.5 ± 98.29a
**LL**	226.01 ± 28.94c	24.00 ± 3.07c	0.21 ± 0.04b	277.75 ± 32.22b	650 ± 91.11c	147.5 ± 29.88b
**LL+MT**	383.21 ± 19.47b	49.26 ± 2.34b	0.51 ± 0.03b	465.5 ± 78.66a	1579 ± 68.15b	390.5 ± 22.82a

### 4.2 Effect of exogenous MT on ROS accumulation in pepper under LL stress

**Figure 2:**
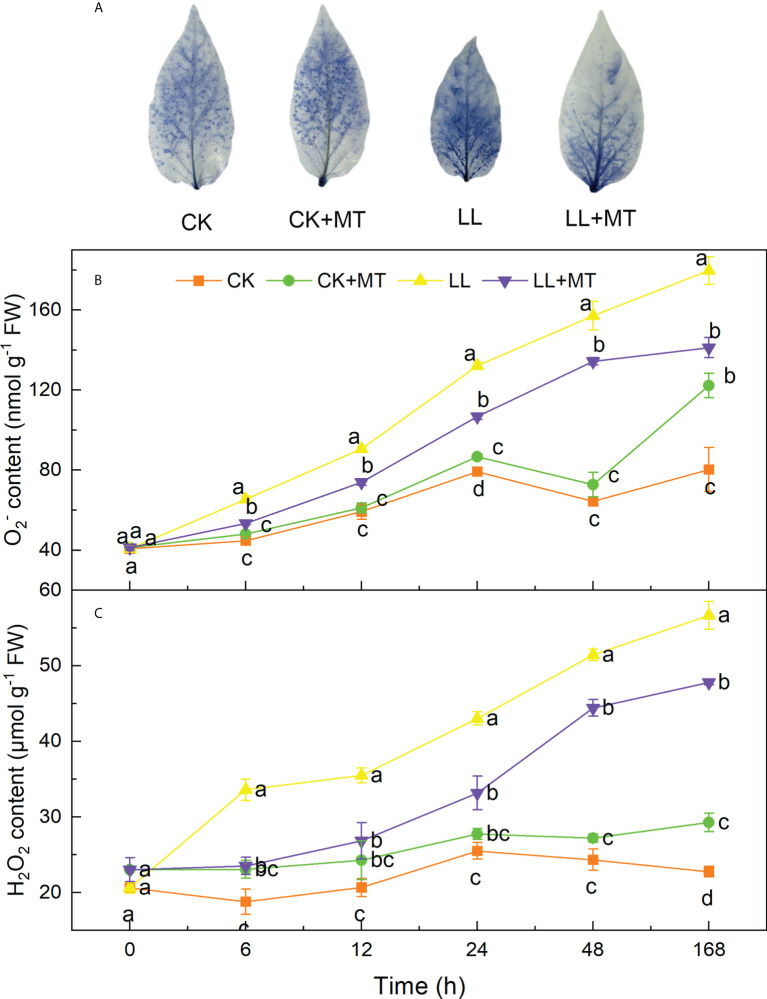
Effect of exogenous MT on the accumulation of ROS in pepper under LL stress. **(A)** Image of NBT staining after treated for 168h, **(B)** superoxide anion ( ${\text{O}}_{2}^{-}1$ ) content, **(C)** Hydrogen peroxide (H_2_O_2_) content. CK (normal temperature and light, 28°C/18°C and 300 μmol photons m^–2^ s^–1^), CK+MT (normal temperature and light, 28°C/18°C and 300 μmol photons m^–2^ s^–1^, 200 μmol L^-1^ melatonin), LL (low–temperature combined with low–light, 15°C/5°C and 100 μmol m^–2^ s^–1^), and LL+MT (low–temperature combined with low–light, 15°C/5°C and 100 μmol m^–2^ s^–1^, 200 μmol L^-1^ melatonin). Vertical bars represent the standard errors of the means (three replicates. Different letters denote statistically significant differences among treatments within same time at a level of *P* < 0.05 by Duncan’s multiple comparison tests.

Histochemical detection of ${\text{O}}_{2}^{-}$ contents in pepper leaves was performed by using NBT staining. As shown in **Figure 2A**, the blue areas and depth of pepper leaves were the highest under LL treatment for 168h. However, this was reversed by MT pretreatment. The blue area was obviously reduced and only spread over half of the leaf. This finding was consistent with that shown in **Figures 2B, C** wherein the ${\text{O}}_{2}^{-}$ and H_2_O_2_ contents significantly accumulated under LL stress, gradually increased over time, and reached highest contents after 168h with 1.24-fold and 2.49-fold of CK, respectively. Both ${\text{O}}_{2}^{-}$ and H_2_O_2_ contents in leaves of seedlings treated under LL were still the highest among four treatments after 6, 12, 24, 48, and 168 h. However, MT pre-spraying reduced the ROS contents of pepper leaves under LL. The contents of ${\text{O}}_{2}^{-}$ and H_2_O_2_ significantly decreased by 18.33, 18.39, 19.24, 14.49 and 21.41%, and 29.95, 24.41, 22.91, 13.60 and 15.72% after 6, 12, 24, 48 and 168h, respectively.

### 4.3 Effect of exogenous MT on soluble sugar, soluble protein, and MDA in pepper leaves under LL stress

**Figure 3:**
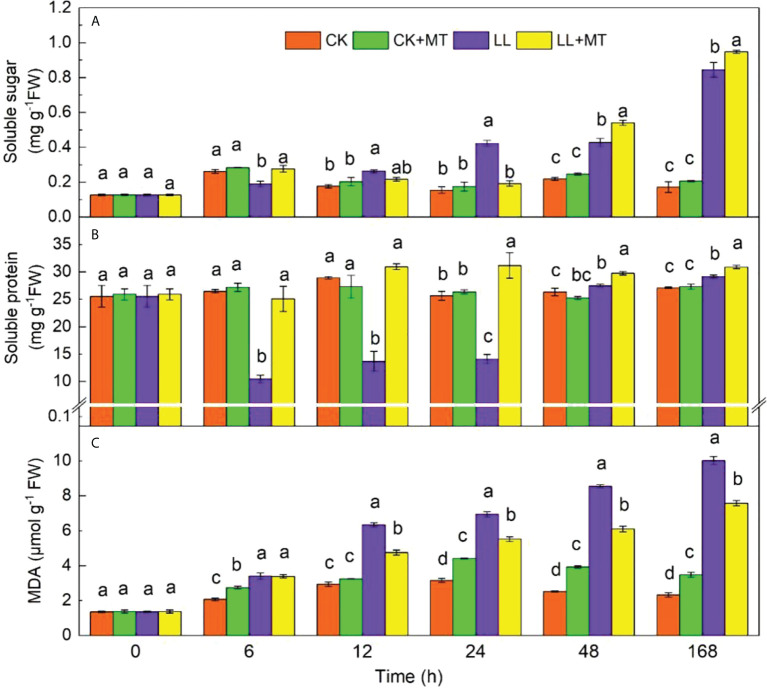
Effect of exogenous MT on **(A)** Soluble sugar content, **(B)** Soluble protein content, **(C)** Malondialdehyde (MDA) content of pepper leaves under LL stress. CK (normal temperature and light, 28°C/18°C and 300 μmol photons m^–2^ s^–1^), CK+MT (normal temperature and light, 28°C/18°C and 300 μmol photons m^–2^ s^–1^, 200 μmol L^-1^ melatonin), LL (low–temperature combined with low–light, 15°C/5°C and 100 μmol m^–2^ s^–1^), and LL+MT (low–temperature combined with low–light, 15°C/5°C and 100 μmol m^–2^ s^–1^, 200 μmol L^-1^ melatonin). Vertical bars represent the standard errors of the means (three replicates). Different letters denote statistically significant differences among treatments within same time at a level of *P* < 0.05 by Duncan’s multiple comparison tests.

The effect of MT on soluble sugar, soluble protein, and MDA is displayed in **Figure 3**. Under LL stress, the soluble sugar content significantly decreased (*P* < 0.05) at the first 6 h and then increased from 12 h to 168 h with contents ranging from 0.262 mg g^-1^ FW to 0.845 mg g^-1^ FW. Meanwhile, exogenous MT significantly enhanced the content of soluble sugar at 6, 48, and 168 h compared with LL, and the highest contents were observed at 168 h (0.948 mg g^-1^ FW, an increase of 12.22%). In terms of soluble protein, LL greatly decreased the soluble protein content at the first 24 h. Then, the content significantly increased after 168 h. The highest soluble protein content was 29.14 mg g^-1^ FW (an increase of 7.57%) at 168 h compared with CK. After the application of MT under LL, the contents of soluble protein significantly increased by 139.97, 125.82, 120.99, 82.71, and 59.47% at 6, 12, 24, 48, and 168 h, respectively However, the MDA contents significantly increased gradually with time under LL stress. Exogenous MT significantly decreased the LL-induced increases in MDA content by 25, 20.50, 28.66, and 24.36% at 12, 24, 48, and 168 h, respectively.

### 4.4 Effect of exogenous MT on antioxidant enzyme activity and the relative expression of related genes in pepper leaves under LL stress

**Figure 4:**
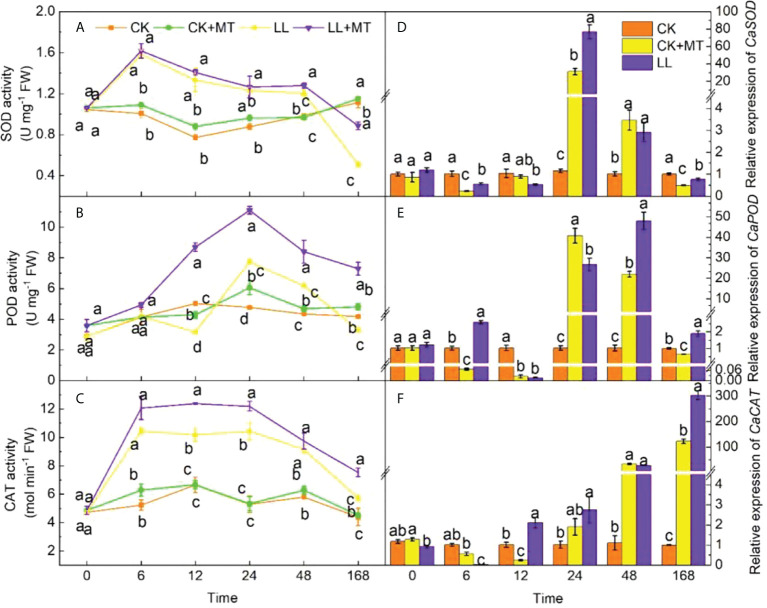
Effect of exogenous MT on antioxidant enzyme activity of pepper leaves under LL stress. **(A)** Superoxide dismutase (SOD) activity, **(B)** Peroxidase (POD) activity, **(C)** Catalase (CAT) activity. **(D)** Relative expression of *CaSOD*, **(E)** Relative expression of *CaPOD*, **(F)** Relative expression of *CaCAT*. CK (normal temperature and light, 28°C/18°C and 300 μmol photons m^–2^ s^–1^), CK+MT (normal temperature and light, 28°C/18°C and 300 μmol photons m^–2^ s^–1^, 200 μmol L^-1^ melatonin), LL (low–temperature combined with low–light, 15°C/5°C and 100 μmol m^–2^ s^–1^), and LL+MT (low–temperature combined with low–light, 15°C/5°C and 100 μmol m^–2^ s^–1^, 200 μmol L^-1^ melatonin). Vertical bars represent the standard errors of the means (three replicates). Different letters denote statistically significant differences among treatments within same time at a level of *P* < 0.05 by Duncan’s multiple comparison tests.

The activities of SOD, POD, and CAT enzymes and the relative expression of *CaSOD*, *CaPOD*, and *CaCAT* under different treatments were investigated (**Figure 4**) in pepper leaves to estimate the effects of MT pre-application. Obviously, when exposed to LL stress, the SOD activity (**Figure 4A**) of pepper leaves with and without MT significantly increased by 57.12, 72.34, 40.17, and 21.81% at 6, 12, 24, and 48 h, respectively, and then significantly decreased by 54.38% at 168 h in contrast to CK. The SOD activity with MT pretreatment showed the same tendency under LL. Notably, the SOD activity increased significantly by 6.27 and 74.26% at 48 and 168h, respectively (*P* < 0.05) compared with LL. LL stress significantly enhanced the POD activity (**Figure 4B**) from 24 h to 48 h and finally decreased by 21.27% at 168 h compared with no stress. Meanwhile, the POD of MT-pretreated seedlings was significantly higher than those of other treatments (CK, CK+MT, and LL) at 12, 24, 48, and 168 h, displaying an increase of 176.09, 43.22, 36.08, and 121.05%, respectively compared with LL. After LL stress, the CAT activity (**Figure 4C**) of seedlings increased during the first 24 h, and then gradually decreased, which was still higher than that of CK with an increase of 53.06-97.22% within 168 h. The pre-application of MT increased CAT activity of pepper seedlings under LL, and showed a significant increase by 21.80, 16.94, and 31.70% at 12, 24, and 168 h, respectively, compared with LL.

**Figures 4D–F**show that compared with CK, LL downregulated the relative expression of *CaSOD*, *CaPOD*, and *CaCAT* during the first 12h. Then, LL upregulated *CaSOD* and *CaPOD* from 24 h to 48 h, finally, downregulated by 50.84 and 35.78% at 168 h. *CaCAT* gradually increased from 24 h to 168 h, which was 127.17-fold higher than that of CK at 168 h. MT spraying showed significant effects on the relative expression of *CaSOD*, *CaPOD*, and *CaCAT* at 168 h under LL stress (increased by 54.00, 193.02, and 147.73%, respectively).

### 4.5 Effect of exogenous MT on AsA-GSH enzyme activity and genes in pepper leaves under LL stress

**Figure 5:**
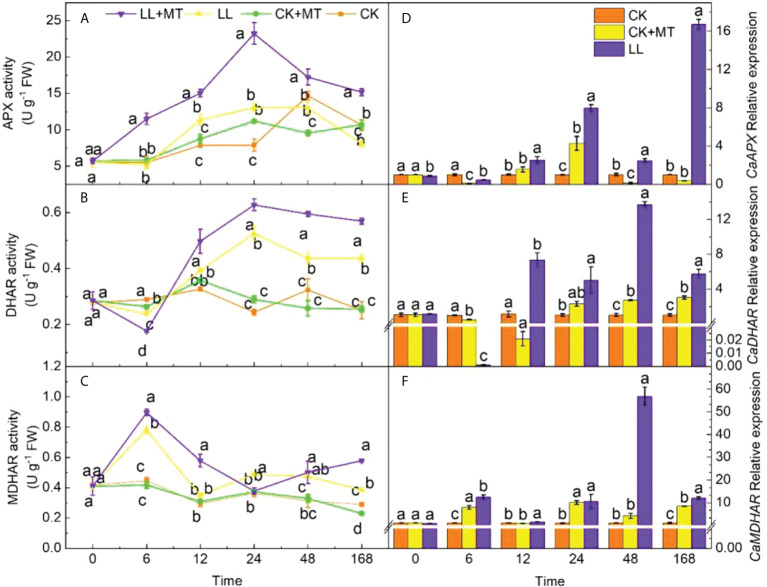
Effect of exogenous MT on ASA-GSH enzyme activity and genes in pepper leaves under LL stress. **(A)** Ascorbate peroxidase (APX) activity, **(B)** Dehydroascorbate reductase (DHAR), **(C)** Monodehydroascorbate reductase (MDHAR) activity. **(D)** Relative expression of *CaAPX*, **(E)** Relative expression of *CaDHAR*, **(F)** Relative expression of *CaMDHAR*. CK (normal temperature and light, 28°C/18°C and 300 μmol photons m^–2^ s^–1^), CK+MT (normal temperature and light, 28°C/18°C and 300 μmol photons m^–2^ s^–1^, 200 μmol L^-1^ melatonin), LL (low–temperature combined with low–light, 15°C/5°C and 100 μmol m^–2^ s^–1^), and LL+MT (low–temperature combined with low–light, 15°C/5°C and 100 μmol m^–2^ s^–1^, 200 μmol L^-1^ melatonin). Vertical bars represent the standard errors of the means (three replicates). Different letters denote statistically significant differences among treatments within same time at a level of *P* < 0.05 by Duncan’s multiple comparison tests.

**Figure 5** shows the changes in the activity levels of enzymes involved in the AsA–GSH cycle of pepper leaves pretreated with MT under LL. Relative to CK, LL stress influenced the activities of enzymes in the AsA–GSH cycle. The activities of APX showed a significant increase at 12 h but decreased at 168 h (declining by 24.10%). The APX activities were significantly enhanced by pretreated with MT, and reached a peak value of 23.24 U g^-1^ FW (increased by 77.89%) at 24 h. Then, the value decreased from 24 h to 168 h, which was still significantly higher than that of LL, and increased by 87.95% after 168 h. LL remarkably suppressed the DHAR activity (**Figure 5B**) in pepper leaves at 6 h but enhanced the activity at 24, 48, and 168 h. The peak value of DHAR activity was 1.16 times higher than that in CK at 24 h. The DHAR activities of seedlings pretreated with MT after LL stress were significantly higher than those with LL at any time except at 6 h. The highest value was observed at 24 h, which was 19.65% higher than that of LL. **Figure 5C** demonstrates that the MDHAR activity significantly increased by 74.43, 41.70, 65.70, and 32.81% at 6, 24, 48, and 168 h under LL stress, respectively. MT application remarkably increased the activity of MDHAR by 14.83, 65.70, and 50.38% at 6, 12, and 168 h, respectively.

Similarly, the relative expression level of *CaAPX* (**Figure 5D**) notably increased at 24 h and decreased at 6, 48 h, and 168 h under LL. However, the relative expression of *CaAPX* in pepper leaves treated with MT increased at 6–168 h compared with LL, and 43.69 times of LL after treated for 168h under LL. The expression of *CaDHAR* (**Figure 5E**) was obviously inhibited by LL treatment at 6 and 12 h; however, it was enhanced at 24 to 168 h and showed significant differences at 6 (decreased by 49.97%) and 48 and 168 h ((increased by 165.08 and 193.60%, respectively). MT application remarkably increased the expression of *CaDHAR* after 12, 24, 48, and168 h, and were 350.38, 1.18, 4.02, and 0.90 times higher than that of LL. The relative expression of *CaMDHAR* (**Figure 5F**) in pepper seedlings was significantly enhanced at 6, 24, and 168 h and was 6.78, 8.77, and 6.87 times higher than that of CK, respectively. After pretreatment with MT under LL, the expression of *CaMDHAR* was dramatically increased by 56.96, 60.89, 12.33, and 41.69% at 6, 12, 48, and 168 h, respectively.

### 4.6 Effect of exogenous MT on AsA-GSH cycle contents in pepper leaves under LL stress

**Figure 6:**
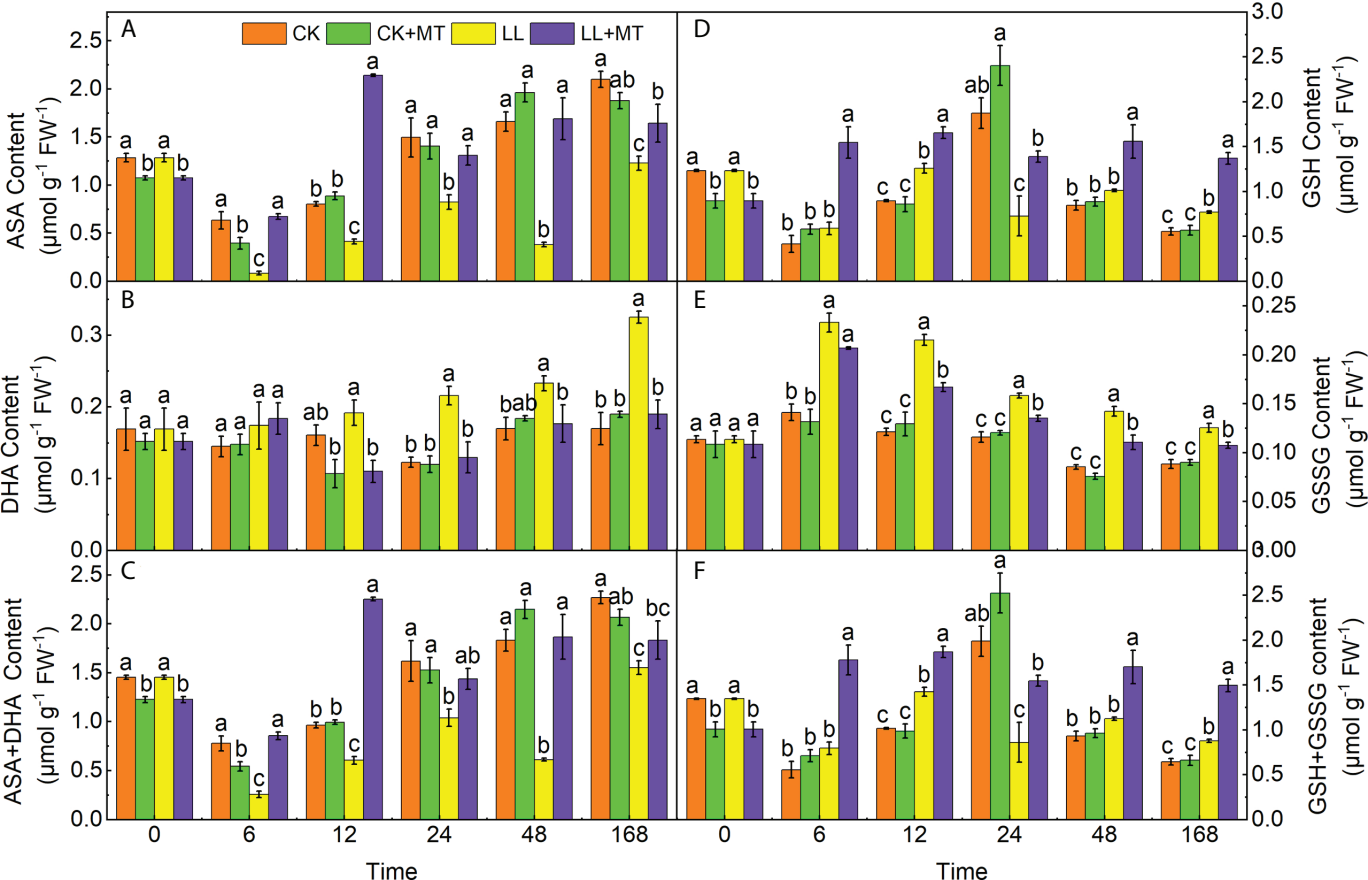
Effect of exogenous MT on ASA-GSH contents in pepper leaves under LL stress. **(A)** Ascorbic acid (AsA) content, **(B)** Dehydroascorbate (DHA) content, (**(C)**AsA + DHA content, **(D)** Glutathione content, **(E)** Oxidized glutathione (GSSG) content, **(F)** GSH + GSSG content. CK (normal temperature and light, 28°C/18°C and 300 μmol photons m^–2^ s^–1^), CK+MT (normal temperature and light, 28°C/18°C and 300 μmol photons m^–2^ s^–1^, 200 μmol L^-1^ melatonin), LL (low–temperature combined with low–light, 15°C/5°C and 100 μmol m^–2^ s^–1^), and LL+MT (low–temperature combined with low–light, 15°C/5°C and 100 μmol m^–2^ s^–1^, 200 μmol L^-1^ melatonin). Vertical bars represent the standard errors of the means (three replicates). Different letters denote statistically significant differences among treatments within same time at a level of *P* < 0.05 by Duncan’s multiple comparison tests.

**Figure 6** demonstrated the changes in the contents of AsA, DHA, AsA+DHA, GSH, GSSG, and GSH+GSSG after MT pre-application under LL in pepper leaves. ASA contents (**Figure 6A**) significantly decreased by 86.71, 48.56, 44.91, 77.10, and 41.53 at 6, 12, 24, 48 and 168 h under LL compared with CK. However, the AsA contents in seedlings treated with MT under LL were significantly enhanced, which were 6.99, 4.17, 0.59, 3.44, and 0.34 times higher at 6, 12, 24, 48 and 168 h than those of LL, respectively. The DHA contents (**Figure 6B**) displayed no significant differences under LL at 6 and 12 h, compared to CK, but significant increases of 75.37, 37.20, and 91.40% were observed at 24, 48, and 168 h. Whereas, MT pre-application significantly declined DHA contents by 42.48, 39.93, 24.13, and 41.57% at 12, 24, 48 and 168 h under LL. The varies of AsA+ DHA contents were similar to ASA contents, which significantly declined by 31.57% after LL stress for 168h compared to CK, while were reversed by pre-adding MT and increased by 18.11% in comparison with LL (**Figure 6C**).

As shown in **Figure 6D**, compared to CK, GSH contents of seedlings under LL stress showed no changes at 6 and 48 h, but significantly decreased by 61.21% at 24h, and slightly increased by 40.23 and 38.66% at 12 and 168h. In contrast, MT dramatically increased GSH contents in pepper under LL after 6, 12, 24, 48, and 168 h, which increased by 162.55, 31.62, 91.06, 53.96, and 77.78%, respectively, compared with LL. Differently, GSSG contents (**Figure 6D**) significantly increased by 64.93, 77.36, 36.55, 66.55, and 42.16% at 6, 12, 24, 48, and 168 h under LL, whereas, these were reversed by MT pre-application, which declined by 11.20, 22.36, 14.62, 22.11, and 14.35%. The changes of GSH + GSSG contents (**Figure 6F**) were similar to GSH contents, compared with CK, declined by 56.67% at 24 h, but enhanced by 36.34% at 168h under LL. While, GSH + GSSG contents in seedlings pre-treated with MT were 1.24, 0.31, 0.79, 0.51, 0.70 times higher than those of LL at 6, 12, 24, 48, and 168 h, respectively.

## 5 Discussion

Low temperature combined with low-light stress is one of the most devastating stresses limiting the growth and development of plants by causing a series of physiological, biochemical, and molecular disorders (Cui et al., 2016; Shu et al., 2016; Bi et al., 2017; Anwar et al., 2020; Bawa et al., 2020; Liu et al., 2020b; Zhang J.F. et al., 2020). As reported previous, LL has adverse effects on plant seedlings and results in significant reduction in growth (Li et al., 2021; Ding et al., 2022; Li et al., 2022). Our results demonstrated that the LL-induced inhibition was associated with significant increases in ROS and changes in antioxidant enzymes in pepper seedlings. MT is a naturally pleiotropic molecule in plants, and is considered to be a plant growth regulator involved in improving plant resistance against environmental stresses (Zhang et al., 2015; Li et al., 2019a; Chen et al., 2020; Yan et al., 2021). The present findings revealed that MT-pre-application treatment significantly influenced the pepper seedling root and antioxidant systems under LL stress.

The root system plays a dominant role in absorbing water and nutrients from the soil or substrate, and reflects whether the plants are stressed. Hussain et al. (2020) indicated that the root surface area and root volume of XD889, XD319, Yu13, and Yu37 maize cultivars were significantly reduced under a low temperature of 15°C. In the present study, the root surface area, root volume, total root length, forks, tips, and crossing of pepper seedlings were all decreased after 168h under LL stress. Recent evidence showed that MT treatment stimulated adventitious and lateral root formation and root elongation (Arnao and Hernandez-Ruiz, 2007; Park and Back, 2012; Sarropoulou et al., 2012; Zhang et al., 2013; Wang et al., 2016). Zhang et al. (2019) found that aluminum-induced inhibition of *Arabidopsis* root length may be associated with the reduced MT caused by *AtSNAT* downregulation, which was reversed by exogenous MT application and endogenous MT synthesis (Zhang et al., 2019). Similarity, a study found that the soybean root growth was increased after supplementation with low concentration of MT (0.1-1.0 μM) under aluminium toxicity, which might be contributed to the enhanced expression of *NSI-X1* and *NSI-X2* involved in MT biosynthesis (Zhang et al., 2017b). Dai et al. (2020) stated that the taproot and lateral root length of rapeseed (*Brassica napus* L.) *cv* Qinyou 8 seedlings irrigated with 100 μM MT for 7 days were promoted under drought, enabling normal water uptake, compared with seedlings without MT. While, no pronounced effects on total root length, root surface area, root diameter, and root volume were found, indicating that MT enhance drought tolerance by improving lateral and taproot growth (Dai et al., 2020). This is inconsistent with the results of our study, wherein the root length, root surface area, tips, forks, and crossing were increased compared with seedlings without MT-spraying by 105.25, 142.86, 67.60, 142.92, and 164.75% (*P* < 0.05), respectively (**Table 1**). The reason for these inconsistent results may be due to different stresses or species. Our findings suggested that MT improved the LL-tolerance of pepper seedlings by alleviating LL-induced root growth limitation, which might be upregulate the key genes in MT biosynthesis in roots.

The plasma membrane is the first site that senses the temperature, and low temperature induced substantially alterations in lipid composition (Lado et al., 2016). Plants are expose to adverse environment throughout their life which generally cause excessive ROS generation causing lipids damage (Hassan et al., 2021). ROS are widely considered to be signaling molecules that are involved in oxidative stress. Although plants possess well-developed antioxidant system and other defense systems, stresses are likely to induce surfeit accumulation of ROS, resulting in membrane lipid peroxidation and reduction of enzyme activity (Heidarvand and Amiri, 2010). In the present study, short time (0-48h) and long time (168h) LL stress significantly increased the ${\text{O}}_{2}^{-}$ and H_2_O_2_ contents (**Figure 2**), causing oxidative stress to seedlings as a result of continuously increased MDA concentration (**Figure 3**), and suggesting critically irreparable lipid peroxidation and severe damage to the plasma membrane. Exogenous substances have been widely used to enhance plant tolerance to LL. However, the role of MT in ROS and antioxidant system in pepper seedlings is still limited. In the present study, pretreated with MT, although the ${\text{O}}_{2}^{-}$ and H_2_O_2_ contents in pepper leaves under LL stress were obviously higher than those under CK, but significantly decreased after 6, 12, 24, 48, and 168h compared with LL, which indicated that exogenous MT effectively alleviated the LL-induced damage in pepper plants. Our results are consistent with those of a previous study that in “Zhongnong 37” cucumber (*Cucumis sativus* L.) sprayed 200μM MT for 5 days during 2-leaf stage, a significant decrease of 246.1% in MDA contents were observed on 6th day under 15/10°C and 95% humidity, in addition, the H_2_O_2_ contents were greatly reduced on the second and fourth day, and the production of ${\text{O}}_{2}^{-}$ was dramatically suppressed, compared to the seedlings without MT treatment (Amin et al., 2022). Membrane stability damage is considered to be a dominant stress caused by low temperature combined with low light as indicated by the accumulations of H_2_O_2_ and ${\text{O}}_{2}^{-}$ . While, MT functions as an antioxidant and a free radical scavenger, its supplementation protects the membrane and maintains membrane stability by scavenging ROS. Zhang et al. (2021) reported that the H_2_O_2_ content displayed a 65.5% increase when cucumber seedlings were exposed to 8°C or 5°C, however, this effect was reversed by application of MT, in which less H_2_O_2_ content increase (37.8%) was detected compared with the seedlings treated with H_2_O (Zhang et al., 2021). Whereas, more evidence proved that the favorable function of MT is not merely participated in ROS scavenging, MT also could strongly induce ROS signaling and desensitize plant ion channels to ROS (Liu et al., 2020a). Generally, plants use increasing ROS generated by stresses to stimulate multiple Ca^2+^ signaling pathways acting upstream or post-translational adaptive mechanisms (Huang et al., 2022). Studies have demonstrated that transcript levels of many calcium signals and stress-related kinases were upregulated in Arabidopsis treated with MT (Weeda et al., 2014). These results affirmed that exogenous MT substantially reduced MDA and ROS accumulation, and the possible role of MT in triggering more efficient ROS signaling, preventing membrane lipid peroxidation and oxidative injury, maintaining membrane stability, (Tan et al., 2007), eventually improving the tolerance to LL.

The accumulation of osmolytes, especially carbohydrate, is an imperative mechanisms in response to stresses in plants (Hassan et al., 2021). The contents of carbohydrate, including soluble sugar and soluble protein, are necessary for plants as a source of energy, and play a crucial role in plant tolerance against stress. Stresses have been shown to change the soluble sugar and soluble protein concentrations to adapt to environmental alternation (Kobylinska et al., 2018). In a previous report, plants accumulated higher osmolyte in the cytosol to prevent the adverse effects of osmotic stress (Latef and He, 2011), and tolerant plant species with good growth performance release high concentrations of osmolytes under stress, which enhance protein function and tissue water contents (Ahanger et al., 2018). Consistently, in our study, LL stress significantly reduced the soluble protein content after stressed for short time (6, 12, and 24h), but increased after 48 and 168h compared with CK. Differently, the soluble sugar content decreased at 6h, but increased at 12, 24, 48, and 168 h, which implied that the soluble protein and soluble sugar contents were changed in response to LL stress. However, the response of soluble sugar response was faster than that of soluble protein in pepper seedlings. The foliar spray of MT maintained higher soluble sugar after 48 and 168h stress, but soluble protein contents for any time under LL stress that kept cell water contents, maintain membrane stability, and improved the plant LL tolerance. Irshad et al. (2021) also found the osmolyte agents (proline, glycine betaine, soluble sugars, and protein) in *Medicago truncatula* were increased by pre-application of 75 μM MT under cold stress (Irshad et al., 2021). Similar results of MT-enhanced osmolytes were found to be induced in alfalfa under low temperature (Liu et al., 2019), grafted *Carya cathayensis* plants under drought (Sharma et al., 2020), and sorghum seedlings exposed to waterlogging (Zhang et al., 2022). Except the regulation of osmotic adjustment, soluble sugar and soluble protein function as energy reservoirs, providing carbon and nitrogen storage for plant growth and key components for cell survival (Singh et al., 1987). Therefore, MT pretreatment was inferred to enhance LL tolerance by effectively maintaining substance biosynthesis and membrane stability caused by improved osmolyte and carbohydrate levels.

ROS induced by LL are closely related to the antioxidant defense system, and are considered as the direct pathway to alleviate the negative impact of ROS. SOD, POD, and CAT are vital enzymes responsible for scavenging ROS in the plant defense system, SOD first catalyzes ${\text{O}}_{2}^{-}$ to H_2_O_2_, and then POD and CAT decompose H_2_O_2_ to H_2_O and O_2_, which present oxidative injury induced by stresses (Anwar et al., 2018). Numerous researches have proved that exogenous MT application enhances the antioxidant defense mechanism against adverse conditions (Ding et al., 2018; Wei et al., 2018; Wang et al., 2020). The SOD, POD, and CAT activities of cucumber seedlings treated with MT were increased by 201.30, 213.92, and 477.63%, respectively under low temperature and high humidity for 4 days. The key enzyme-encoding genes (*CsSOD*, *CsPOD*) were dramatically upregulated at day 2, 4, and 6, and the highest expression level was obtained at day 4, increasing by 69.25 and 95.33% in comparison with those of seedlings without MT. However, *CsCAT* increased from day 0 to day 6 (Amin et al., 2022). In our experiment, MT pretreatment exerted slight effects on the SOD, POD, and CAT activities under control condition. Pepper seedlings exposed to LL stress significantly enhanced the activities of SOD after 6-48h, CAT after 6-168h, POD after 24-48h, but all activities in seedlings pretreated with MT increased from 0h to 168h except SOD at 168h (**Figures 3A–C**). At the same time, the maximum relative expression levels of *CaSOD*, *CaPOD*, and *CaCAT* were obtained in MT groups under LL at 24, 48, and 168h, respectively. The reduction in ROS accumulation triggered by MT improved plant performance under stress, which was positively correlated with the strengthened activities of antioxidant enzymes. Sun et al. (2018) also proposed that the upregulated expression of Cu/Zn and Fe-SOD genes caused by MT caused the increase of SOD activity, mitigating oxidative stress and enhancing tolerance against cold (Sun et al., 2018). These results implied that MT plays a synergistic role in LL resistance.

ROS in plants exhibit homeostasis under suitable conditions, whereas excess ROS content generated by abiotic stress causes the oxidative injury (Xu et al., 2019). Consequently, to cope with the oxidative injury triggered by stresses, plants initiate enzymatic antioxidant defense mechanisms and nonenzymatic mechanisms. Most importantly, it has to mention that AsA and GSH are indispensable nonenzymatic antioxidants located in plant cell organelles and cytoplasm. In the current study, H_2_O_2_ and MDA accumulated in pepper seedling leaves under LL stress and the NBT staining result showed a dark blue color (**Figure 2**), which suggested that cell membrane lipid peroxidation already occurred. The dominant pathway to clear H_2_O_2_ in plants is through the AsA-GSH cycle, and APX, DHAR, MDHAR, AsA, and GSH levels are enhanced in LL-stressed seedlings to conquer reduced oxidative stress (MDA, H_2_O_2_, ${\text{O}}_{2}^{-}$ ). However, the capacity of ROS scavenging was limited, especially in severe stress. The pretreatment of MT significantly boosts the antioxidant system by improving antioxidant enzyme activities and expression of related genes (Sun et al., 2019). Rice plants with over-expressed dehydroascorbate reductase gene (*OsDHAR1*) exhibited enhanced ascorbate levels, photosynthetic ability, and membrane stability (Kim et al., 2022). Similar trends were reported in tomato seedlings, wherein the GR and APX activities as well as AsA contents were significantly increased after exposure to 0.1mM MT for 3 days under drought (Liu et al., 2015). Li et al. (2017) also revealed that under salinity conditions, the activities of main enzymes including APX, DHAR, and MDHAR were significantly decreased, but the decrease was mitigated when *Citrullus lanatus* L. was treated with 150 μM MT. Moreover, AsA and GSH were dramatically higher in plants treated with MT than those treated with H_2_O, resulting highest ratios of GSH/GSSH and AsA/DHA (Li et al., 2017). Additionally, in MT-deficient tomato seedlings, increasing ROS accumulation and server lipid peroxidation were observed which was associated with significantly declined endogenous MT under chilling, however, MT-supplementation improved this, meanwhile, reduced ratios of AsA/DHA in MT deficient seedlings displayed low antioxidant enzymes capacity were reversed by exogenous MT (Wang et al., 2020). These results were well agreed with our findings, wherein MT promoted APX, DHAR, and MDHAR activities, as well as AsA and GSH contents, and inhibited the increased contents of DHA and GSSH caused by LL. Furthermore, a experiment conducted by Li et al. (2019b) demonstrated that MT positively upregulated the expression of *CsAPX* in tea plant under stresses of cold, salt, and drought stresses, leading to increased ROS elimination (Li et al., 2019b). The expressions of *CsAPX*, *CsMDHAR*, *CsDHAR*, and *CsGR* in cucumber were all upregulated by MT under low-temperature and high-humidity stress (Amin et al., 2022). This result was consistent with our findings in which *CaAPX*, *CaDHAR*, and *CaMDHAR* were upregulated by pre-application of MT after 12, 24, 48, and 168h LL stress (**Figure 6**). These findings indicated that MT could improve cell redox homeostasis and cell membrane stability by inducing the activities of enzymatic and non-enzymatic antioxidant defense system, regulating AsA-GSH cycle, and upregulating the expression of antioxidant enzymes genes.

## 6 Conclusions

**Figure 7:**
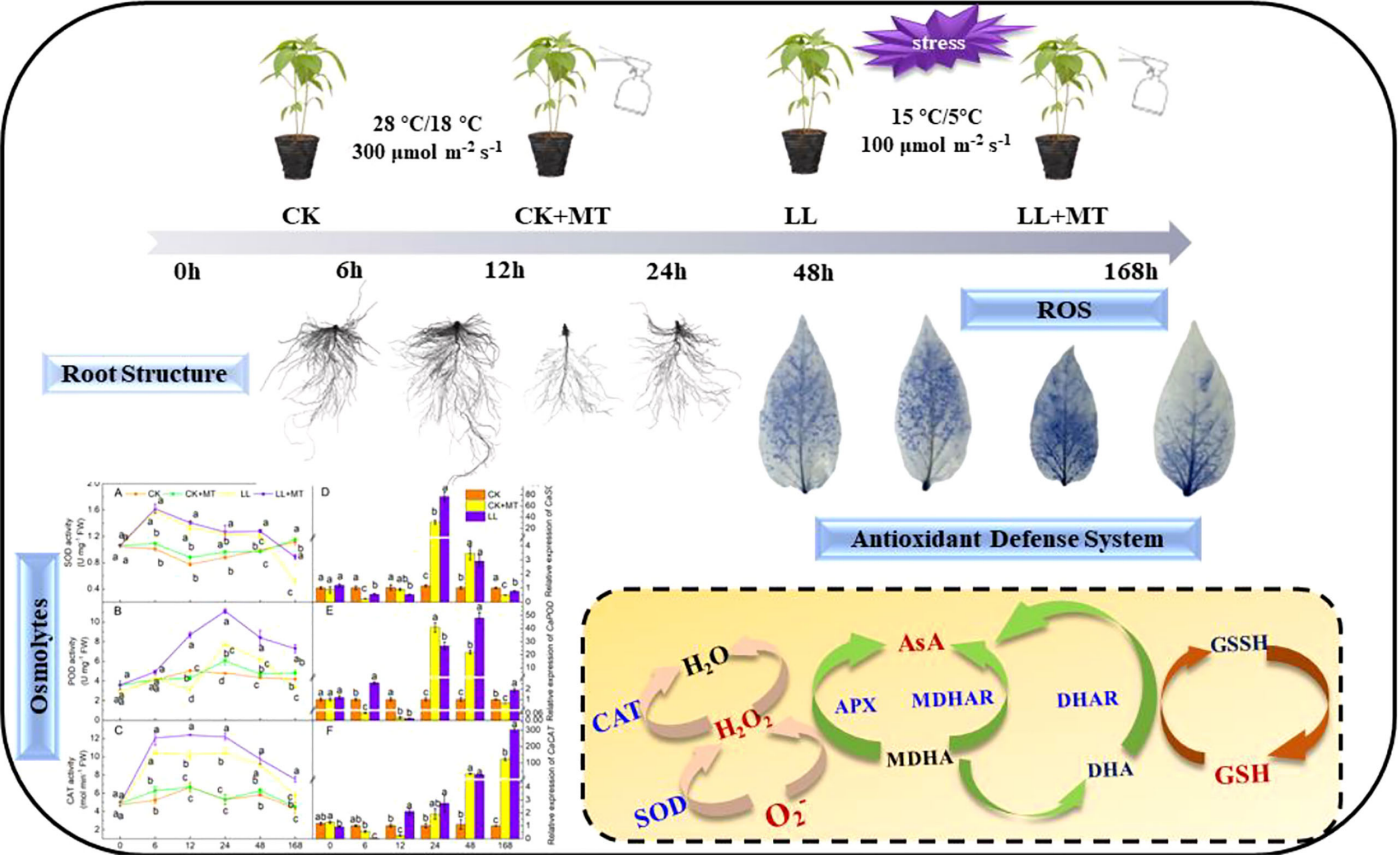
Schematic overview of melatonin enhanced low-temperature combined with low-light tolerance of pepper (*Capsicum annuum* l.) seedlings by regulating root growth, antioxidant defense system, and osmotic adjustment.

LL stress inhibited pepper root growth and caused oxidative stress by increasing accumulations of ROS levels and lipid peroxidation degrees in pepper seedlings. However, MT reversed the phenomena and enhanced LL tolerance in pepper seedlings. MT Preapplication improved the root morphology structure and cell structure. Additionally, MT application rescued the LL-induced oxidative stress by exerting a synergistic effect that regulated of osmotic adjustment (soluble sugar and soluble protein) and a series of antioxidant enzymes (SOD, POD, CAT, APX, DHAR, MDHAR) and substances (AsA, DHA, GSH, and GSSH), as well as upregulating the expression of *CaSOD*, *CaPOD*, *CaCAT*, *CaAPX*, *CaDHAR*, *CaMDHAR*. Therefore, MT pretreatment was inferred to enhance LL tolerance by effectively maintaining substance biosynthesis, membrane stability, and ROS scavenging (**Figure 7**).

## Data availability statement

The original contributions presented in the study are included in the article/**Supplementary Material**. Further inquiries can be directed to the corresponding author.

## Author contributions

JL, JX, JY, JLy, and JFZ: conceptualization. JL, DD, and NL: methodology. JL, DD, NL, FG: software. JL, JFZ, DD, NL, JZ, YY, and TN: formal analysis, investigation. JL: writing—original draft preparation. JL and JX: writing—review and editing. JX: resources, supervision, project administration, and funding acquisition. All authors contributed to the article and approved the submitted version.

## Funding

The work was financially supported by the Natural Science Foundation of Gansu Province (20JR10RA515), Scientific Research Start-up Funds for Openly-Recruited Doctors (GAU-KYQD-2018-35), National Natural Science Foundation of China (32072657), and the Special Fund for Technical System of Melon and Vegetable Industry of Gansu Province (GARS-GC-1), Special Fund for Sci. & Tech. Innovation and Development Guided by Gansu Province (2018ZX-02), National Key Research and Development Program of China (2016YFD0201005), and The writers extremely acknowledge all the authors, the editors and the reviewers for the precious and valuable comments and suggestions, which have greatly improved the article.

## Conflict of interest

The authors declare that the research was conducted in the absence of any commercial or financial relationships that could be construed as a potential conflict of interest.

## Publisher’s note

All claims expressed in this article are solely those of the authors and do not necessarily represent those of their affiliated organizations, or those of the publisher, the editors and the reviewers. Any product that may be evaluated in this article, or claim that may be made by its manufacturer, is not guaranteed or endorsed by the publisher.

## Abbreviations

APX, ascorbate peroxidase; AsA, reduced ascorbic acid; AsA-GSH, reduced ascorbic acid- reduced glutathione; CAT, catalase; DHA, dehydroascorbate; DHAR, dehydroascorbate reductase; GR, glutathione reductase; GSH, reduced glutathione; GSSG, oxidized glutathione; H_2_O_2_, hydrogen peroxide; low-temperature combined with low-light (LL); MDA, malondialdehyde; MDHAR, monodehydroascorbate reductase; MT, melatonin; NBT, nitroblue tetrazolium; ${\text{O}}_{2}^{-}$ , superoxide anion radical; POD, peroxidase; qRT-PCR, quantitative real-time polymerase chain reaction; ROS, reactive oxygen species; SOD, superoxide dismutase.

## References

[B1] Ahammed G. J. Wu M. Wang Y. Yan Y. Mao Q. Ren J.. (2020). Melatonin alleviates iron stress by improving iron homeostasis, antioxidant defense and secondary metabolism in cucumber. Sci. Hortic. 265, 109205. doi: 10.1016/j.scienta.2020.109205

[B2] Ahammed G. J. Xu W. Liu A. Chen S. (2019). Endogenous melatonin deficiency aggravates high temperature-induced oxidative stress in solanum lycopersicum l. Environ. Exp. Bot. 161, 303–311. doi: 10.1016/j.envexpbot.2018.06.006

[B3] Ahanger M. A. Alyemen i. Wijaya L. Alamri S. A. Alam P. Ashraf M.. (2018). Potential of exogenously sourced kinetin in protecting solanum lycopersicum from NaCl-induced oxidative stress through up-regulation of the antioxidant system, ascorbate-glutathione cycle and glyoxalase system. PloS One 13 (9), e0202175. doi: 10.1371/journal.pone.0202175 30180173 PMC6122799

[B4] Ahmad S. Kamran M. Ding R. Meng X. Wang H. Ahmad I.. (2019). Exogenous melatonin confers drought stress by promoting plant growth, photosynthetic capacity and antioxidant defense system of maize seedlings. Peerj 7, e7793. doi: 10.7717/peerj.7793 31616591 PMC6791350

[B5] Amin B. Atif. M. J. Meng H. Ali M. Li S. Alharby H. F.. (2022). Melatonin rescues photosynthesis and triggers antioxidant defense response in *Cucumis sativus* plants challenged by low temperature and high humidity. Front. Plant Sci. 13. doi: 10.3389/fpls.2022.855900 PMC9094117 35574101

[B6] Anwar A. Wang J. Yu X. C. He C. X. Li Y. S. (2020). Substrate application of 5-aminolevulinic acid enhanced low-temperature and weak-light stress tolerance in cucumber (*Cucumis sativus* l.). Agronomy-Basel 10 (4), 472. doi: 10.3390/agronomy10040472

[B7] Anwar A. Yan Y. Liu Y. Li Y. Yu X. (2018). 5-aminolevulinic acid improves nutrient uptake and endogenous hormone accumulation, enhancing low-temperature stress tolerance in cucumbers. Int. J. Mol. Sci. 19 (11), 3379. doi: 10.3390/ijms19113379 PMC6275039 30380613

[B8] Arnao M. B. Hernandez-Ruiz J. (2007). Melatonin promotes adventitious- and lateral root regeneration in etiolated hypocotyls of *Lupinus albus* l. J. Pineal Res. 42 (2), 147–152. doi: 10.1111/j.1600-079X.2006.00396.x 17286746

[B9] Balabusta M. Szafranska K. Posmyk M. M. (2016). Exogenous melatonin improves antioxidant defensein cucumber seeds (*Cucumis sativus* l.) germinated under chilling stress. Front. Plant Sci. 7. doi: 10.3389/fpls.2016.00575 PMC4848318 27200048

[B10] Barrero-Sicilia C. Silvestre S. Haslam R. P. Michaelson L. V. (2017). Lipid remodelling: unravelling the response to cold stress in arabidopsis and its extremophile relative *Eutrema salsugineum* . Plant Sci. 263, 194–200. doi: 10.1016/j.plantsci.2017.07.017 28818375 PMC5567406

[B11] Bawa G. Feng L. Y. Chen G. P. Chen H. Hu Y. Pu T.. (2020). Gibberellins and auxin regulate soybean hypocotyl elongation under low light and high-temperature interaction. Physiol. Plantarum 170 (3), 345–356. doi: 10.1111/ppl.13158 32588443

[B12] Bi H. G. Liu P. P. Jiang Z. S. Ai X. Z. (2017). Overexpression of the rubisco activase gene improves growth and low temperature and weak light tolerance in cucumis sativus. Physiol. Plantarum 161 (2), 224–234. doi: 10.1111/ppl.12587 28543370

[B13] Bradford M. M. (1976). A rapid and sensitive method for the quantitation of microgram quantities of protein utilizing the principle of protein-dye binding. Anal. Biochem. 72, 248–254. doi: 10.1006/abio.1976.9999 942051

[B14] Campos C. N. Avila R. G. Dazio De Souza K. R. Azevedo L. M. Aalves J. D. (2019). Melatonin reduces oxidative stress and promotes drought tolerance in young *Coffea arabica* l. plants. Agr. Water Manage 211, 37–47. doi: 10.1016/j.agwat.2018.09.025

[B15] Chen L. Fan J. Hu Z. Huang X. Amombo E. Liu A.. (2017). Melatonin is involved in regulation of bermudagrass growth and development and response to low k^+^ stress. Front. Plant Sci. 8, 2038. doi: 10.3389/fpls.2017.02038 29234342 PMC5712302

[B16] Chen L. Liu L. T. Lu B. Ma T. T. Jiang D. Li J.. (2020). Exogenous melatonin promotes seed germination and osmotic regulation under salt stress in cotton (*Gossypium hirsutum* l.). PloS One 15 (1), e0228241. doi: 10.1371/journal.pone.0228241 32004326 PMC6994006

[B17] Chen L. Zhao Y. Xu S. Zhang Z. Xu Y. Zhang J.. (2018). *OsMADS57* together with *OsTB1* coordinates transcription of its target *OsWRKY94* and *D14* to switch its organogenesis to defense for cold adaptation in rice. New Phytol. 218 (1), 219–231. doi: 10.1111/nph.14977 29364524 PMC5873253

[B18] Cui L. R. Zou Z. R. Zhang J. Zhao Y. Y. Yan. F. (2016). 24-epibrassinoslide enhances plant tolerance to stress from low temperatures and poor light intensities in tomato (*Lycopersicon esculentum* mill.). Funct. Integr. Genomic. 16 (1), 29–35. doi: 10.1007/s10142-015-0464-x 26337714

[B19] Dai L. Li J. Harmens H. Zheng X. Zhang C. (2020). Melatonin enhances drought resistance by regulating leaf stomatal behaviour, root growth and catalase activity in two contrasting rapeseed (*Brassica napus* l.) genotypes. Plant Physiol. Bioch. 149, 86–95. doi: 10.1016/j.plaphy.2020.01.039 32058897

[B20] Debnath B. Hussain M. Irshad M. Mitra S. Li M. Liu S.. (2018). Exogenous melatonin mitigates acid rain stress to tomato plants through modulation of leaf ultrastructure, photosynthesis and antioxidant potential. Molecules 23 (2), 388. doi: 10.3390/molecules23020388 PMC6017902 29439491

[B21] Dey S. Biswas A. Huang S. Li D. Liu L. Deng Y.. (2021). Low temperature effect on different varieties of corchorus capsularis and corchorus olitorius at seedling stage. Agronomy-Basel 11 (12), 2547. doi: 10.3390/agronomy11122547

[B22] Ding D. Li J. Xie J. Li N. Bakpa E. P. Han K.. (2022). Exogenous zeaxanthin alleviates low temperature combined with low light induced photosynthesis inhibition and oxidative stress in pepper (*Capsicum annuum* l.) plants. Curr. Issues Mol. Biol. 44 (6), 2453–2471. doi: 10.3390/cimb44060168 35735609 PMC9221838

[B23] Ding F. Wang G. Zhang S. (2018). Exogenous melatonin mitigates methyl viologen-triggered oxidative stress in poplar leaf. Molecules 23 (11), 2852. doi: 10.3390/molecules23112852 PMC6278511 30400163

[B24] Eom K. C. Jung P. ,. K. Choi S. H. Kim T. W. Yoo S. Y. Park S. H.. (2010). Water requirement of red pepper in different growth stages. Korean J. Soil Sci. Fertilizer 43 (6), 844–847.

[B25] Hassan M. U. Chattha M. U. Khan I. Chattha M. B. Barbanti L. Aamer M.. (2021). Heat stress in cultivated plants: nature, impact, mechanisms, and mitigation strategies-a review. Plant Biosyst. 155 (2), 211–234. doi: 10.1080/11263504.2020.1727987

[B26] Hattori A. Migitaka H. Iigo M. Itoh M. Yamamoto K. Ohtani-Kaneko R.. (1995). Identification of melatonin in plants and its effects on plasma melatonin levels and binding to melatonin receptors in vertebrates. Biochem. Mol. Biol. Int. 35 (3), 627–634. 7773197

[B27] Heidarvand L. Amiri R. M. (2010). What happens in plant molecular responses to cold stress? Acta Physiol. Plant 32 (3), 419–431. doi: 10.1007/s11738-009-0451-8

[B28] Huang X. Tanveer M. Min Y. Shabala S. (2022). Melatonin as a regulator of plant ionic homeostasis: implications for abiotic stress tolerance. J. Exp. Bot. erac224 doi: 10.1093/jxb/erac224 35640481

[B29] Hussain H. A. Men S. Hussain S. Zhang Q. Ashraf U. Anjum S. A.. (2020). Maize tolerance against drought and chilling stresses varied with root morphology and antioxidative defense system. Plants-Basel 9 (6), 720. doi: 10.3390/plants9060720 PMC7356637 32517168

[B30] Imran M. Khan M. A. Shahzad R. Bilal S. Khan M. Yun B. W.. (2021). Melatonin ameliorates thermotolerance in soybean seedling through balancing redox homeostasis and modulating antioxidant defense, phytohormones and polyamines biosynthesis. Molecules 26 (17), 5116. doi: 10.3390/molecules26175116 34500550 PMC8434054

[B31] Irshad A. Ur Rehman R. N. Kareem H. A. Yang P. Hu T. (2021). Addressing the challenge of cold stress resilience with the synergistic effect of rhizobium inoculation and exogenous melatonin application in medicago truncatula. Ecotox. Environ. Safe 226, 112816. doi: 10.1016/j.ecoenv.2021.112816 34597844

[B32] Kim Y. S. Park S. I. Kim J. J. Shin S. Y. Kwak S. S. Lee C. H.. (2022). Over-expression of dehydroascorbate reductase improves salt tolerance, environmental adaptability and productivity in *Oryza sativa* . Antioxidants 11 (6), 1077. doi: 10.3390/antiox11061077 35739975 PMC9220092

[B33] Kobylinska A. Borek S. Posmyk M. M. (2018). Melatonin redirects carbohydrates metabolism during sugar starvation in plant cells. J. Pineal Res. 64 (4), e12466. doi: 10.1111/jpi.12466 29292521

[B34] Kong F. Deng Y. Zhou B. Wang G. Wang Y. Meng Q. (2014). A chloroplast-targeted DnaJ protein contributes to maintenance of photosystem II under chilling stress. J. Exp. Bot. 65 (1), 143–158. doi: 10.1093/jxb/ert357 24227338 PMC3883286

[B35] Lado J. Rodrigo M. J. Lopez-Climent M. Gomez-Cadenas A. Zacarias L. (2016). Implication of the antioxidant system in chilling injury tolerance in the red peel of grapefruit. Postharvest Biol. Tec. 111, 214–223. doi: 10.1016/j.postharvbio.2015.09.013

[B36] Latef A. He C. X. (2011). Arbuscular mycorrhizal influence on growth, photosynthetic pigments, osmotic adjustment and oxidative stress in tomato plants subjected to low temperature stress. Acta Physiol. Plant 33 (4), 1217–1225. doi: 10.1007/s11738-010-0650-3

[B37] Li H. Chang J. Chen H. Wang Z. Gu X. Wei C.. (2017). Exogenous melatonin confers salt stress tolerance to watermelon by improving photosynthesis and redox homeostasis. Front. Plant Sci. 8. doi: 10.3389/fpls.2017.00295 PMC5331065 28298921

[B38] Li J. Ding D. Li N. Xie J. Yu J. Lyv J.. (2022). Melatonin enhances the low-temperature combined low-light tolerance of pepper (*Capsicum annuum* l.) seedlings by regulating photosynthesis, carotenoid, and hormone metabolism. Environ. Exp. Bot. 199, 104868. doi: 10.1016/j.envexpbot.2022.104868

[B39] Li J. Liu J. Zhu T. Zhao C. Li L. Chen M. (2019a). The role of melatonin in salt stress responses. Int. J. Mol. Sci. 20 (7), 1735. doi: 10.3390/ijms20071735 PMC6479358 30965607

[B40] Liu Y.-S. Geng J.-C. Sha X.-Y. Zhao Y.-X. Hu T.-M. Yang P.-Z. (2019). Effect of rhizobium symbiosis on low-temperature tolerance and antioxidant response in alfalfa (*Medicago sativa* l.). Front. Plant Sci. 10. doi: 10.3389/fpls.2019.00538 PMC6503086 31114600

[B41] Liu J. Shabala S. Zhang J. Ma G. Chen D. Shabala L.. (2020a). Melatonin improves rice salinity stress tolerance by NADPH oxidase-dependent control of the plasma membrane k^+^ transporters and k^+^ homeostasis. Plant Cell Environ. 43 (11), 2591–2605. doi: 10.1111/pce.13759 32196121

[B42] Liu J. Wang B. Li Y. Huang L. Zhang Q. Zhu H.. (2020b). RNA Sequencing analysis of low temperature and low light intensity-responsive transcriptomes of zucchini (*Cucurbita pepo* l.). Sci. Hortic. 265. doi: 10.1016/j.scienta.2020.109263

[B43] Liu J. Wang W. Wang L. Sun Y. (2015). Exogenous melatonin improves seedling health index and drought tolerance in tomato. Plant Growth Regul. 77 (3), 317–326. doi: 10.1007/s10725-015-0066-6

[B44] Livak K. J. Schmittgen T. D. (2001). Analysis of relative gene expression data using real-time quantitative PCR and the 2(-delta delta C(T)) method. Methods 25 (4), 402–408. doi: 10.1006/meth.2001.1262 11846609

[B45] Li X. Wei J. Scott E. R. Liu J. Guo S. Li Y.. (2018). Exogenous melatonin alleviates cold stress by promoting antioxidant defense and redox homeostasis in *Camellia sinensis* l. Molecules 23 (1), 165. doi: 10.3390/molecules23010165 PMC6017414 29342935

[B46] Li J. Xie J. Yu J. Lyv J. Bakpa E. P. Zhang X.. (2021). Transcriptome sequence and physiological analysis revealed the roles of carotenoids and photosynthesis under low temperature combined with low-light stress on pepper (*Capsicum annuum* l.). Photosynthetica 59 (1), 24–36. doi: 10.32615/ps.2020.083

[B47] Li J. Yang Y. Sun K. Chen Y. Chen X. Li X. (2019b). Exogenous melatonin enhances cold, salt and drought stress tolerance by improving antioxidant defense in tea plant (*Camellia sinensis* (L.) o. kuntze). Molecules 24 (9), 1826. doi: 10.3390/molecules24091826 PMC6539935 31083611

[B48] Mclay L. K. Nagarajan-Radha V. Green M. P. Jones T. M. (2018). Dim artificial light at night affects mating, reproductive output, and reactive oxygen species in drosophila melanogaster. J. Exp. Zool. Part. A. 329 (8-9), 419–428. doi: 10.1002/jez.2164 29733537

[B49] Meng Z. Lu T. Zhang G. Qi M. Tang W. Li L.. (2017). Photosystem inhibition and protection in tomato leaves under low light. Sci. Hortic. 217, 145–155. doi: 10.1016/j.scienta.2017.01.039

[B50] Moustafa-Farag M. Mahmoud A. Arnao M. B. Sheteiwy M. S. Dafea M. Soltan M.. (2020). Melatonin-induced water stress tolerance in plants: Recent advances. Antioxidants 9 (9), 809. doi: 10.3390/antiox9090809 PMC7554692 32882822

[B51] Nagasuga K. Murai-Hatano M. Kuwagata T. (2011). Effects of low root temperature on dry matter production and root water uptake in rice plants. Plant Prod. Sci. 14 (1), 22–29. doi: 10.1626/pps.14.22

[B52] Ou L. J. Wei G. Zhang Z. Q. Dai X. Z. Zou X. X. (2015). Effects of low temperature and low irradiance on the physiological characteristics and related gene expression of different pepper species. Photosynthetica 53 (1), 85–94. doi: 10.1007/s11099-015-0084-7

[B53] Page V. Bloesch R. M. Feller U. (2012). Regulation of shoot growth, root development and manganese allocation in wheat (*Triticum aestivum*) genotypes by light intensity. Plant Growth Regul. 67 (3), 209–215. doi: 10.1007/s10725-012-9679-1

[B54] Park S. Back K. (2012). Melatonin promotes seminal root elongation and root growth in transgenic rice after germination. J. Pineal Res. 53 (4), 385–389. doi: 10.1111/j.1600-079X.2012.01008.x 22640001

[B55] Ren J. Ye J. Yin L. Li G. Deng X. Wang S. (2020). Exogenous melatonin improves salt tolerance by mitigating osmotic, ion, and oxidative stresses in maize seedlings. Agronomy-Basel 10 (5), 663. doi: 10.3390/agronomy10050663

[B56] Sarropoulou V. Dimassi-Theriou K. Therios I. Koukourikou-Petridou M. (2012). Melatonin enhances root regeneration, photosynthetic pigments, biomass, total carbohydrates and proline content in the cherry rootstock PHL-c (*Prunus avium x prunus cerasus*). Plant Physiol. Bioch. 61, 162–168. doi: 10.1016/j.plaphy.2012.10.001 23127522

[B57] Sharma A. Wang J. Xu D. Tao S. Chong S. Yan D.. (2020). Melatonin regulates the functional components of photosynthesis, antioxidant system, gene expression, and metabolic pathways to induce drought resistance in grafted carya cathayensis plants. Sci. Total Environ. 713, 136675. doi: 10.1016/j.scitotenv.2020.136675 32019031

[B58] Sharma A. Zheng B. (2019). Melatonin mediated regulation of drought stress: Physiological and molecular aspects. Plants-Basel 8 (7), 190. doi: 10.3390/plants8070190 PMC6681211 31248005

[B59] Shi H. Qian Y. Tan D. X. Reiter R. J. He C. (2015). Melatonin induces the transcripts of CBF/DREB1s and their involvement in both abiotic and biotic stresses in arabidopsis. J. Pineal Res. 59 (3), 334–342. doi: 10.1111/jpi.12262 26182834

[B60] Shu S. Tang Y. Y. Yuan Y. H. Sun J. Zhong M. Guo S. R. (2016). The role of 24-epibrassinolide in the regulation of photosynthetic characteristics and nitrogen metabolism of tomato seedlings under a combined low temperature and weak light stress. Plant Physiol. Bioch. 107, 344–353. doi: 10.1016/j.plaphy.2016.06.021 27362298

[B61] Singh N. K. Bracker C. A. Hasegawa P. M. Handa A. K. Buckel S. Hermodson M. A.. (1987). Characterization of osmotin: a thaumatin-like protein associated with osmotic adaptation in plant cells. Plant Physiol. 85 (2), 529–536. doi: 10.1104/pp.85.2.529 16665731 PMC1054289

[B62] Sun Y. Liu Z. Lan G. Jiao C. Sun Y. (2019). Effect of exogenous melatonin on resistance of cucumber to downy mildew. Sci. Hortic. 255, 231–241. doi: 10.1016/j.scienta.2019.04.057

[B63] Sun L. Li X. Wang Z. Sun Z. Zhu X. Liu S.. (2018). Cold priming induced tolerance to subsequent low temperature stress is enhanced by melatonin application during recovery in wheat. Molecules 23 (5), 1091. doi: 10.3390/molecules23051091 PMC6100458 29734723

[B64] Tang C. Xie J. Lv J. Jing L. Zhang J. Wang C.. (2021). Alleviating damage of photosystem and oxidative stress from chilling stress with exogenous zeaxanthin in pepper (*Capsicum annuum* l.) seedlings. Plant Physiol. Bioch. 162, 395–409. doi: 10.1016/j.plaphy.2021.03.010 33740679

[B65] Tan D.-X. Mancheste r. Helton P. Reiter R. J. (2007). Phytoremediative capacity of plants enriched with melatonin. Plant Signal. Behav. 2 (6), 514–516. doi: 10.4161/psb.2.6.4639 19704544 PMC2634354

[B66] Wang Q. An B. Wei Y. Reiter R. J. Shi H. Luo H.. (2016). Melatonin regulates root meristem by repressing auxin synthesis and polar auxin transport in arabidopsis. Front. Plant Sci. 7, 1882. doi: 10.3389/fpls.2016.01882 28018411 PMC5156734

[B67] Wang M. Zhang S. Ding F. (2020). Melatonin mitigates chilling-induced oxidative stress and photosynthesis inhibition in tomato plants. Antioxidants 9 (3), 218. doi: 10.3390/antiox9030218 PMC7139585 32155702

[B68] Weeda S. Zhang N. Zhao X. Ndip G. Guo Y. Buck G. A.. (2014). Arabidopsis transcriptome analysis reveals key roles of melatonin in plant defense systems. PloS One 9 (3), e93462. doi: 10.1371/journal.pone.0093462 24682084 PMC3969325

[B69] Wei Z. Gao T. Liang B. Zhao Q. Ma F. Li C. (2018). Effects of exogenous melatonin on methyl viologen-mediated oxidative stress in apple leaf. Int. J. Mol. Sci. 19 (1), 316. doi: 10.3390/ijms19010316 PMC5796259 29361738

[B70] Xu W. Cai S.-Y. Zhang Y. Wang Y. Ahammed G. J. Xia X.-J.. (2016). Melatonin enhances thermotolerance by promoting cellular protein protection in tomato plants. J. Pineal Res. 61 (4), 457–469. doi: 10.1111/jpi.12359 27484733

[B71] Xu Y. Charles M. T. Luo Z. Mimee B. Tong Z. Veronneau P.-Y.. (2019). Ultraviolet-c priming of strawberry leaves against subsequent mycosphaerella fragariae infection involves the action of reactive oxygen species, plant hormones, and terpenes. Plant Cell Environ. 42 (3), 815–831. doi: 10.1111/pce.13491 30481398

[B72] Yamori N. Levine C. P. Mattson N. S. Yamori W. (2022). Optimum root zone temperature of photosynthesis and plant growth depends on air temperature in lettuce plants. Plant Mol. Biol. 2022, 1–11. doi: 10.1007/s11103-022-01249-w 35169910

[B73] Yan F. Y. Wei H. M. Ding Y. F. Li W. W. Liu Z. H. Chen L.. (2021). Melatonin regulates antioxidant strategy in response to continuous salt stress in rice seedlings. Plant Physiol. Bioch. 165, 239–250. doi: 10.1016/j.plaphy.2021.05.003 34082330

[B74] Zeng W. Mostafa S. Lu Z. Jin B. (2022). Melatonin-mediated abiotic stress tolerance in plants. Front. Plant Sci. 13. doi: 10.3389/fpls.2022.847175 PMC9125191 35615125

[B75] Zhang X. Feng Y. Jing T. Liu X. Ai X. Bi H. (2021). Melatonin promotes the chilling tolerance of cucumber seedlings by regulating antioxidant system and relieving photoinhibition. Front. Plant Sci. 12. doi: 10.3389/fpls.2021.789617 PMC8695794 34956288

[B76] Zhang J. Li D. Wei J. Ma W. Kong X. Rengel Z.. (2019). Melatonin alleviates aluminum-induced root growth inhibition by interfering with nitric oxide production in arabidopsis. Environ. Exp. Bot. 161, 157–165. doi: 10.1016/j.envexpbot.2018.08.014

[B77] Zhang J. F. Li J. Xie J. M. Yu J. H. Dawuda M. M. Lyv J.. (2020). Changes in photosynthesis and carotenoid composition of pepper (Capsicum annuum l.) in response to low-light stress and low temperature combined with low-light stress. Photosynthetica 58 (1), 125–136. doi: 10.32615/ps.2019.175

[B78] Zhang J. Shi Y. Zhang X. Du H. Xu B. Huang B. (2017a). Melatonin suppression of heat-induced leaf senescence involves changes in abscisic acid and cytokinin biosynthesis and signaling pathways in perennial ryegrass (*Lolium perenne* l.). Environ. Exp. Bot. 138, 36–45. doi: 10.1016/j.envexpbot.2017.02.012

[B79] Zhang T. Shi Z. Zhang X. Zheng S. Wang J. Mo J. (2020). Alleviating effects of exogenous melatonin on salt stress in cucumber. Sci. Hortic. 262. doi: 10.1016/j.scienta.2019.109070

[B80] Zhang N. Sun Q. Zhang H. Cao Y. Weeda S. Ren S.. (2015). Roles of melatonin in abiotic stress resistance in plants. J. Exp. Bot. 66 (3), 647–656. doi: 10.1093/jxb/eru336 25124318

[B81] Zhang R. Yue Z. Chen X. Wang Y. Zhou Y. Xu W.. (2022). Foliar applications of urea and melatonin to alleviate waterlogging stress on photosynthesis and antioxidant metabolism in sorghum seedlings. Plant Growth Regul. 97 (2), 429–438. doi: 10.1007/s10725-021-00705-9

[B82] Zhang J. Zeng B. Mao Y. Kong X. Wang X. Yang Y.. (2017b). Melatonin alleviates aluminium toxicity through modulating antioxidative enzymes and enhancing organic acid anion exudation in soybean. Funct. Plant Biol. 44 (10), 961–968. doi: 10.1071/fp17003 32480624

[B83] Zhang N. Zhao B. Zhang H.-J. Weeda S. Yang C. Yang Z.-C.. (2013). Melatonin promotes water-stress tolerance, lateral root formation, and seed germination in cucumber (Cucumis sativus l.). J. Pineal Res. 54 (1), 15–23. doi: 10.1111/j.1600-079X.2012.01015.x 22747917

[B84] Zhao H. Ye L. Wang Y. Zhou X. Yang J. Wang J.. (2016). Melatonin increases the chilling tolerance of chloroplast in cucumber seedlings by regulating photosynthetic electron flux and the ascorbate-glutathione cycle. Front. Plant Sci. 7. doi: 10.3389/fpls.2016.01874 PMC5138187 27999581

[B85] Zhu J. Zhang K.-X. Wang W.-S. Gong W. Liu W.-C. Chen H.-G.. (2015). Low temperature inhibits root growth by reducing auxin accumulation *via* ARR1/12. Plant Cell Physiol. 56 (4), 727–736. doi: 10.1093/pcp/pcu217 25552473

