# Supplementary material for: Melatonin enhanced low-temperature combined with low-light tolerance of pepper (Capsicum annuum L.) seedlings by regulating root growth, antioxidant defense system, and osmotic adjustment
Source: Front Plant Sci. 2022 Sep 28;13:998293. doi: 10.3389/fpls.2022.998293 (PMC9554354; doi:10.3389/fpls.2022.998293)
Supplement: Supplementary file 1 [file Table_1.docx]

**Table S1.** The sequences of primers used for the qRT-PCR

| Gene name | Sequence (5′-3′) | Products length (bp) | GenBank accession number |
| --- | --- | --- | --- |
| *CaSOD* | F:GTGAGCCTCCAAAGGGTTCTCTTG | 127 | AF036936.2:35–721 |
|  | R: AAACCAAGCCACACCCAACCAG |  |  |
| *CaPOD* | F: GCCAGGACAGCAAGCCAAGG | 131 | FJ596178.1:68–1042 |
|  | R: TGAGCACCTGATAAGGCAACCATG |  |  |
| *CaCAT* | F: TTAACGCTCCCAAGTGTGCTCATC | 116 | NM_001324674.1:72–1550 |
|  | R: GGCAGGACGACAAGGATCAAACC |  |  |
| *CaAPX* | F: TGTTGTTGCTGTTGAGGTCACTGG | 98 | AF442387.1:24–887 |
|  | R: CATCTGGTAACCGCCCTTCCTTTG |  |  |
| *CaDHAR* | F: CCATATGTCAAAGGGCAGAA | 110 | KJ950368.1:1:64-702 |
|  | R: CTTTCAGGCACACTCCACTT |  |  |
| *CaMDHAR* | F: TACTTCTACTCCCGTGCCTT | 153 | XM_016687442.1:79-1380 |
|  | R: GAGGAATGCACCAACGATCT |  |  |
| *Actin* | F: GTCCTTCCATCGTCCACAGG | 133 | XM_016722297.1 |
|  | R: GAAGGGCAAAGGTTCACAACA |  |  |
